# Feasibility of an ED-to-Home Intervention to Engage Patients: A Mixed-Methods Investigation

**DOI:** 10.5811/westjem.2017.2.32570

**Published:** 2017-04-19

**Authors:** Jessica R. Schumacher, Barbara J. Lutz, Allyson G. Hall, Jesse M. Pines, Andrea L. Jones, Phyllis Hendry, Colleen Kalynych, Donna L. Carden

**Affiliations:** * University of Florida, Department of Emergency Medicine, Gainesville, Florida; † University of North Carolina-Wilmington, College of Health and Human Services, School of Nursing, Wilmington, North Carolina; ‡ University of North Carolina-Wilmington, College of Health and Human Services, School of Social Work, Wilmington, North Carolina; § University of Alabama at Birmingham, Department of Health Services Administration, Birmingham, Alabama; ¶ The George Washington University School of Medicine, Department of Emergency Medicine and Health Policy & Management, Washington, DC; || University of Florida, College of Medicine, Department of Emergency Medicine, Jacksonville, Florida

## Abstract

**Introduction:**

Older, chronically ill patients with limited health literacy are often under-engaged in managing their health and turn to the emergency department (ED) for healthcare needs. We tested the impact of an ED-initiated coaching intervention on patient engagement and follow-up doctor visits in this high-risk population. We also explored patients’ care-seeking decisions.

**Methods:**

We conducted a mixed-methods study including a randomized controlled trial and in-depth interviews in two EDs in northern Florida. Participants were chronically ill older ED patients with limited health literacy and Medicare as a payer source. Patients were assigned to an evidence-based coaching intervention (n= 35) or usual post-ED care (n= 34). Qualitative interviews (n=9) explored patients’ reasons for ED use. We assessed average between-group differences in patient engagement over time with the Patient Activation Measure (PAM) tool, using logistic regression and a difference-in-difference approach. Between-group differences in follow-up doctor visits were determined. We analyzed qualitative data using open coding and thematic analysis.

**Results:**

PAM scores fell in both groups after the ED visit but fell significantly more in “usual care” (average decline −4.64) than “intervention” participants (average decline −2.77) (β=1.87, p=0.043). There were no between-group differences in doctor visits. Patients described well-informed reasons for ED visits including onset and severity of symptoms, lack of timely provider access, and immediate and comprehensive ED care.

**Conclusion:**

The coaching intervention significantly reduced declines in patient engagement observed after usual post-ED care. Patients reported well-informed reasons for ED use and will likely continue to make ED visits unless strategies, such as ED-initiated coaching, are implemented to help vulnerable patients better manage their health and healthcare.

## INTRODUCTION

Patient engagement is central to many health policy initiatives. 1–5 Engaged patients make more informed healthcare decisions, avert health crises and incur lower healthcare costs.^6^,^7^ Interventions to increase patient engagement increase the use of preventive care, reduce hospital-based care and improve outcomes.^6^,^8^–^12^ The Patient Activation Measure SF® (PAM) is a way to quantify patient engagement, which is defined as patients’ knowledge, skills and confidence in managing their health and healthcare and the interventions that promote healthy behaviors. 13 Coaching interventions increase PAM scores, reduce hospital use, and improve medication and chronic disease self-management but have not been tested in the ED.^8^,^9^,^12^

Although ED use is increasing in older adults, those with limited health literacy represent a particularly high-risk group who are often under-engaged in managing their health and frequently turn to the ED for care. 14–18 Strategies aimed at engaging these patients at the critical ED juncture may help them stay engaged, better manage their health and avert future health crises. We tested the impact of a coaching intervention on patient engagement and follow-up doctor visits in chronically ill, older ED patients with limited health literacy. Because efforts to help patients manage their health are more effective if they align with patients’ perspectives,^8^,^19^ we also explored reasons for ED use in this high-risk population.

## METHODS

### Study Design

We conducted a randomized controlled trial (RCT) comparing an ED-to-home intervention (“intervention”) to usual post-ED care (“usual care”) on patient engagement and follow-up doctor visits and in-depth interviews exploring patients’ healthcare-seeking decisions. The study was conducted from July 2013 to August 2014.

### Study Setting

The intervention was tested in two communities. Site 1 ED (90,000 visits/year) is a tertiary referral center serving a community of 250,000 and a White (62%) and African-American (28%) population with various payers (40% public, 36% private). Site 2 ED (89,000 visits/year) is a tertiary referral center serving a metropolitan area of one million and African-American (59%), White (33%), publicly insured (44%) and uninsured (24%) patients.

### Study Population

Older, chronically ill patients with limited health literacy insured by Medicare scheduled for ED discharge were eligible for study inclusion (Figure).

### Study Protocol

#### Recruitment

The university institutional review board approved the study at both sites. Study procedures are outlined (Figure). Random assignment using a random number generator was provided to research associates (RAs) who determined patient eligibility by screening the ED electronic health record (EHR). RAs were blinded to assignment until baseline survey completion.

**Population Health Research Capsule**

What do we already know about this issue?

Coaching interventions increase patient engagement, improve medication and disease self-management and reduce hospital use but have not been tested in the ED.

What was the research question?

Can an ED-initiated coaching intervention increase patient engagement in older ED patients with limited health literacy?

What was the major finding of the study?

The ED-initiated coaching intervention significantly reduced declines in patient engagement observed after usual post-ED care.

How does this improve population health?

ED-initiated coaching interventions hold promise for helping high-risk and hard-to reach patients better manage their health and healthcare.

#### Health Literacy Screening

Following screening and informed consent, patients completed the Rapid Estimate of Adult Literacy in Medicine (REALM).^20^,^21^ REALM is valid in diverse racial/ethnic groups 21 and older adults. 22 Categories include adequate (≥61 words correct; grade level ≥ 9) and limited health literacy (<61 words correct; grade level 0–8).

#### Intervention

The ED-to-home intervention was modeled on the Care Transitions Intervention^SM^ (CTI), an evidence-based program to increase patient engagement and reduce 30-day readmissions and healthcare costs in hospitalized patients. 23 Trained coaches from community area agencies on aging administered the intervention. Coaches helped patients 1) schedule follow-up doctor visits; 2) recognize disease worsening; 3) reconcile medications; and 4) communicate with providers.^10^,^23^,^24^ Coaches visited patients’ homes within three days of ED discharge, called three times over the ensuing month, and engaged patients by helping them set achievable goals.

#### Usual Care

Usual post-ED care included written and verbal discharge instructions and advice to follow up with a provider.

#### In-depth Interviews

Based on site, assignment and date of ED visit, a purposive sample (n=11; 6 “intervention,” 5 “usual care”) was invited to interview, and 9 agreed. Questions emphasized reasons for ED visit and access to post-ED care (Supplementary Appendix S1).

### Data Sources

#### Baseline Survey

Participants completed a baseline ED survey to record PAM score, sociodemographic (age, gender, race), socioeconomic (education, employment, payer status) and health-related factors (self-rated health, number of chronic conditions).

#### Follow-Up Telephone Survey

Participants were called by the University Survey Center within 31–60 days of the ED visit to determine follow-up PAM score and doctor visits using Medicare Current Beneficiary Access-to-Care Survey items. 25 The survey was administered using best practices (e.g., 10 call attempts, rotating call attempts, refusal conversion). 26

#### Patient Activation Measure

We used the 13-item PAM^27^,^28^ to assess engagement including patients’ knowledge, skills, and confidence in managing their health and healthcare. Degrees of agreement with statements, such as “When all is said and done, I am the person who is responsible for managing my health condition” and “Taking an active role in my own health care is the most important factor in determining my health and ability to function,” are scored on a 0–100 point scale. The lowest scores suggest a person does not understand their role in healthcare, while the highest levels indicate greater activation and proactive, healthy behaviors. The PAM is previously published and valid in older, chronically ill patients with limited health literacy.^29^,^30^

#### In-depth Interviews

Interviews (60–90 minutes) were conducted in patients’ homes, audio-recorded and transcribed verbatim.

### Outcome Measures

Outcomes included between-group differences in PAM scores and self-reported doctor visits. In-depth interviews identified factors influencing healthcare-seeking decisions.

### Power Calculation

We conducted a pre-pilot power analysis based on an increase in primary care visits from 30–80% within 10 days of hospital discharge at one study site using the identical coaching intervention in hospitalized patients. Thirty-five participants in both groups were needed to detect similar differences in post-ED visit follow-up with power (1-β) of 80% and alpha of 0.05.

### Quantitative Analysis

We conducted between-group comparisons in sociodemographic, socioeconomic, health status and doctor visits using chi-square and analysis of variance for categorical and continuous measures, respectively. We used an intention-to-treat approach for all analyses.

We assessed between-group differences in PAM scores between the baseline and follow-up time points in two ways. In our primary analysis, we assessed mean PAM score differences over time between the “intervention” and “usual care” groups using unadjusted linear regression. We then assessed between-group PAM score differences over time using a difference-in-differences (DID) approach that accounted for differential between-group loss to follow-up using inverse probability weighting. Inverse probability weighting adjusts for bias due to missing data by giving more weight to patients who resemble those lost to follow-up. 31 The DID approach ensures that background trends in outcomes unrelated to the program are not responsible for treatment effects by comparing outcomes in the treatment group to a group experiencing the same background trends but not exposed to the program. To account for the fact that patient measurements within a site were more likely to be similar than measurements between sites, all models were estimated with standard errors clustered by site. We conducted analyses using Stata v.13, with significance at p<0.05.

### Qualitative Analysis

Interview transcripts were read independently by three members of the research team including qualitative methods experts and a health service researcher. Using thematic and constant comparative analysis, we coded data using procedures described by Charmaz. 32–35 Team members wrote memos to document and record study findings and track methodological, theoretical, and substantive decisions made during the analysis to ensure rigor of data analysis and interpretation. We used open coding to identify concepts important to participants, and provisional themes were presented to the entire team for feedback and verification. Codes were reviewed, discussed and arranged into wider thematic structures to make meaning of participant narratives. 36

## RESULTS

Of 170 patients consenting to health literacy screening, 71 had limited health literacy and were eligible for inclusion (36 “intervention,” 35 “usual care”). All agreed to participate and 69 patients completed baseline ED surveys. Only baseline PAM scores were significantly different between “intervention” and “usual care” groups, respectively (64.0 ±16.9, 60.1±15.1, p=0.03) (Table 1).

Forty of 65 patients able to respond, completed the follow-up telephone survey (61%, 23 “intervention,” 17 “usual “care” [Table 1; Figure]). Patients with lower baseline PAM scores (odds ratio=0.92, 95% CI=0.86–0.98) were less likely to complete the follow-up telephone survey.

Seventy-three percent of “intervention” patients (76% at Site 1; 70% at Site 2) completed coaching. Median time from ED-to-home visit was 2.5 days (range 1–12 days). The home visit lasted approximately 60 minutes, and each of the coaching phone calls lasted about 15 minutes.

### Quantitative Findings

#### Coaching Impact on Patient Engagement

PAM scores fell in both groups after the ED visit but fell significantly more in “usual care” than “intervention” participants (−4.64 and −2.77, respectively; unadjusted linear regression, β=1.87, p=0.043) (Table 2). This finding remained statistically significant after inverse probability weighting to account for loss to follow-up (DID=1.96, t=23.42, p=0.027).

#### Follow-Up Doctor Visits

Most patients (61%) did not report a doctor visit within two weeks of the ED visit (Table 1). “Intervention” participants were more likely to report a follow-up within four weeks of ED visit (74% vs. 65%, respectively, p=0.53).

### Qualitative Findings

Nine interviews (5 “intervention,” 4 “usual care”) were conducted: 5 from Site 1 (4 female, 3 “intervention”) and 4 from Site 2 (3 female; 2 “intervention”). Participant ages ranged from 62–86 years, and all were African American with more than one chronic condition.

#### Patient Engagement and Decision to Seek ED Care

When participants decided to visit the ED, they were highly engaged and motivated to address their health concern. Decisions to visit the ED were well-thought out and driven by *individual characteristics*, including the nature and severity of symptoms, personal advice and prior *healthcare system experiences*. Representative quotes are described below.

#### Individual Characteristics

Unremitting pain and history of similar symptoms factored heavily into patients’ decisions to use the ED. Pain and at least one other precipitating factor (e.g., history of similar symptoms, advice of trusted sources, including providers, friends, family) led to uncertainty, fear, and a decision to seek emergency care for all (9/9) participants.

[2–4]: “I had a pain at the end of my spine and that hip bone that joined together …I had to go. I can’t stand pain no way. And that’s why I ended up going to emergency.”

[1–2]: “I was in a lot of pain for one thing. And I had, beforehand, had a blood clot. So I didn’t know if another one had come back or not, so I thought maybe I needed to go to the emergency room.”

Another participant sought ED care because of history of hyperglycemia.

[1–5]: “I can tell when my sugar goes up because I get really dizzy. The emergency room was the best place to go because if I went to a primary doctor, they were going to send me to an emergency room anyway.”

Participants often considered the advice of family, friends, or healthcare providers.

[2–2]: “I couldn’t straighten up, stand up, or sit down without having cramps. A friend of mine called the ambulance for me, and I was then rushed to the [ED].”

#### Healthcare System Experiences

Decisions to seek ED care were influenced by patients’ prior healthcare system experiences. Perceptions of availability of care in the provider’s office versus ED care were important. For most (6/9), obtaining a same-day appointment or speaking with a provider was not possible even if the patient believed the need was urgent. Patients’ perceptions that the ED provides comprehensive care led (3/9) participants to seek emergency treatment.

A participant with chest tightness and blood pressure of 235/96 [1–4]: “I went to [my doctor], but he was filled up. So I called him and asked him could I come back? And he said no. It would probably 4:00 or 5:00 if I got seen then. I said, “Well, I’m going to have to go to the emergency room because I’m sick. I feel bad.”

[2–4] [My] doctor would [not be able to see me at] “that particular time of day,” so [I] “just went on to emergency.”

Symptoms when providers’ offices were closed influenced decisions to seek ED care. The following participant developed what she thought were minor symptoms on a weekend and decided to wait for symptoms to subside. Her son convinced her to seek immediate care.

[2–1]: “And so I felt like something was sitting on my chest. So I got up and I got ready, and I went on to church. And [the pain] was still there and then I said, “Ah, it’ll go.” So I didn’t go [to the ED]. My son came and he said, ‘Either you let me take you [to the fire station around the corner], or I’ll call 911.’ That I didn’t want, so I said, ‘Well, okay, come and take me around there.’ The [fire fighter] said, ‘Well, we can’t let you go with it…You’re having chest pain.’ I said, ‘Yes, sir, but it feels like gas.’ He said, ‘Well, that’s what heart attacks are like. They are mostly like gas, but then you’re having a heart attack.’ So, I agreed for them to take me to the emergency room because I really wanted to know what it was.”

For participants attending large community-based clinics (health department or Veterans Affairs), timely contact with a provider usually was not possible. Appointments were viewed as a strategy to “maintain” health through check-ups and refilling prescriptions rather than addressing a healthcare concern. For these participants, ED care was considered a reasonable option.

[2–2]: “If I had called [my primary care clinic] to tell them I had cramps they would have given me an appointment two or three months down the line… As a matter of fact, after I went [to the ED in June] and called for a [follow-up] appointment, the earliest appointment they could give me was in August.”

In contrast to perceived lack of availability of timely outpatient care, participants perceived ED care as immediate and comprehensive because of staffing and availability of ancillary and specialty referral services.

[1–3]: “In the ER they get right on it…They won’t let me sit back and die. I know they are coming randomly and checking everything and they know what my levels are. I’m not dying because they would be in there.”

[1–4]: “I figured they had more equipment to do testing over [in the ED] than my primary care doctor did. They can do everything at once.”

Participants’ relationships with their doctor also heavily influenced healthcare-seeking decisions. If the provider was familiar with them and helped them navigate the system, participants were more likely to contact their provider with health concerns and questions about where to seek care. One participant described a partnership with her provider:

[1–1]: “[She] explained things and she insisted that you do things that you know you needed to do.” This patient reported their provider was attentive to the participant’s concerns, and arranged appointments for follow-up tests and regular screenings.

#### Intervention Impact on Patient Engagement

Three of five “intervention” patients indicated the coaching intervention helped them stay involved in their healthcare by increasing their understanding of chronic disease symptoms, appropriate use of medications and follow-up care. They appreciated having someone to call if they had questions. Patients reported receiving help with other needed services.

[2–2]: “Knowing you could call someone, and you don’t have to go through these channels with them. It makes you feel comfortable; not as stressful – there is this person that you know you can call and say ‘this is what is going on’…and they could do something to help you.”

Coaches also helped participants access other community services including transportation [1–2]:“because transportation is my biggest problems.”

## DISCUSSION

Patient engagement has been called the blockbuster drug of the century. When patients are more engaged, they have better health outcomes and lower healthcare costs. 1–5 Unfortunately, interventions aimed at engaging ED patients to help them better manage their health and healthcare have been largely ignored. This study is novel because it focused on chronically ill, older ED patients with limited health literacy, a high-risk population that is often under-engaged in their health and rely on the ED during a health crisis. 14 To our knowledge, this is the first study to assess the impact of an ED-initiated coaching intervention on patient engagement in a vulnerable ED population. The study documented three observations regarding patient engagement. First, chronically ill patients with limited health literacy are most engaged at ED presentation as assessed by PAM scores. Second, engagement falls in the weeks after the ED visit in all patients. Third, the ED-to-home “intervention” significantly reduced the post-ED fall in patient engagement relative to “usual care.”

Baseline PAM scores were higher in this study than reported in a nationally representative sample of Medicare beneficiaries (64.6 versus 63.4 previously reported) 29 but fell to a mean of 61.0 in the weeks following the ED visit. Engagement is highest at ED presentation – a finding that is consistent with prior work that demonstrates patients use the ED when they feel their condition is emergent and are too worried about their condition to seek care in other settings.^16^, ^37^ Our in-depth interviews are consistent with these findings and provide insight into the critical individual and health-system factors that contribute to patients’ decisions to use the ED, which include the onset and severity of symptoms, advice of trusted sources, inability to gain timely access to a provider, and perception that the ED provides immediate, comprehensive care.

We were unable to identify studies where interventions designed to increase patient engagement led to decreased PAM scores. In this study, the observed decline in PAM scores may have occurred because the baseline survey was conducted in the ED. It is likely patients become engaged to take actions about their health when their symptoms suggest something is wrong that requires emergent attention. Although highly engaged in their health during the health crisis, patients’ engagement may decline as the crisis resolves without further health system contact. 38 Importantly, the ED-to-home intervention blunted the decline in post-ED PAM scores by approximately two points. The fact that similar longer-term PAM score changes are associated with improvements in physical activity, medication adherence, self-management knowledge, and functional health suggest the clinical significance of these findings. 38–40

Coaches helped participants advocate for themselves to schedule timely follow-up doctor visits but did not make appointments for patients. Most patients did not see a provider within two weeks of ED visit, and between-group differences were not observed. It is possible that even if ED patients attempted follow-up, they were unable to obtain an appointment because of barriers previously described 41 and noted by our participants. Indeed, a recent systematic review noted that interventions designed to improve post-ED follow-up have variable effectiveness depending on the capacity and willingness of the local primary care network to accommodate ED patients. 42 It is also possible that although this intervention had a significant impact on patient engagement, it did not motivate individuals to attend follow-up doctor visits if they were not already inclined to do so.

## LIMITATIONS

Among this study’s limitations was the small pilot-sample size. Despite the small sample, statistically significant between-group differences in patient engagement were detected and the mixed-methods design allowed us to confirm quantitative findings through in-depth interviews. Second, the pre-pilot increase in follow-up doctor visits observed in hospitalized patients was not detected. Our study may have been underpowered to detect smaller but potentially clinically meaningful increases in post-ED outpatient follow-up visits. In addition, patients may have attempted but were unsuccessful in scheduling post-ED outpatient follow-up visits because of busy clinic schedules. This intervention was conducted in two EDs and the results may not generalize to other settings. In addition, PAM scores fluctuate over time and this study captures only two time points. 7 However, the RCT design and DID approach suggests the observed between-group differences were a true “intervention” effect rather than chance occurrence. Finally, the PAM is proprietary, is not available for use without a license and may not be practical as a clinical tool in the time-sensitive, ED setting.

## CONCLUSION

Patient engagement in chronically ill older patients with limited health literacy was highest at the time of an ED encounter and fell in the following weeks. Qualitative findings confirm and extend these findings, demonstrating that patients’ decisions to visit the ED are the result of engaged decision-making. Our data suggest that an ED initiated coaching intervention reduced the degree of disengagement in healthcare after the ED visit. According to the National Quality Forum, “improved management of transitions of care into and out of the ED has the potential to improve person-centered care, quality, and cost efficiency.” 43 Policy makers and health system managers should consider ED-initiated interventions like this to improve ED-to-home transitions and engagement in high-risk and hard-to-reach ED populations.

## Supplementary Information



## Figures and Tables

**Figure:**
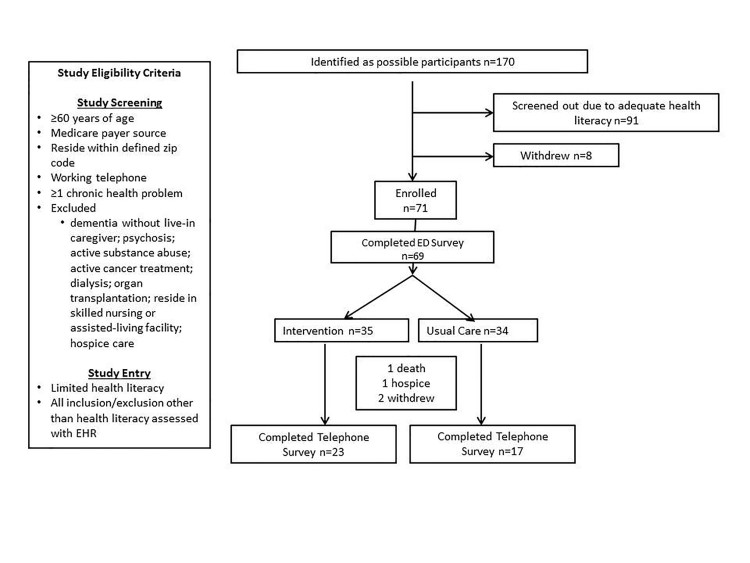
Recruitment procedures. Consolidated standards of reporting trials (CONSORT) flow diagram displaying progress of all participants through the trial.

**Table 1:** Participant characteristics at time of random group assignment and at the time of the follow-up survey.

	Participant characteristics at baseline (n=69)				Participant characteristics at follow-up (n=40)			
		
	Overall (n=69)	Intervention (n=35)	Usual care (n=34)	p-value	Overall (n=40)	Intervention (n=23)	Usual care (n=17)	p-value
Mean age ± SD	72.6 ± 8.8	72.0 ± 8.3	73.2 ± 9.4	0.55	71.5 ± 7.8	70.7 ± 7.2	72.5 ± 8.8	0.47
Gender, n (%)				0.92				0.24
Male	30 (43)	15 (43)	15 (44)		16 (40)	11 (48)	5 (29)	
Female	39 (57)	20 (57)	19 (56)		24 (60)	12 (52)	12 (71)	
Non-white, n (%)				0.52				0.69
Yes	53 (77)	28 (80)	25 (74)		34 (85)	20 (87)	14 (82)	
No	16 (23)	7 (20)	9 (26)		6 (15)	3 (13)	3 (18)	
Self-rated health, n (%)				0.10				0.13
Excellent; very good; good	40 (60)	23 (70)	17 (50)		25 (66)	16 (76)	9 (53)	
Fair or poor	27 (40)	10 (30)	17 (50)		13 (34)	5 (24)	8 (47)	
Mean chronic conditions count ± SD	3.9 ± 1.7	3.9 ± 1.5	3.9 ± 1.9	0.89	3.7 ± 1.5	4.0 ± 1.7	3.3 ± 1.2	0.22
Emergency severity index**				0.22				0.08
High acuity	32 (48)	19 (56)	13 (41)		20 (51)	14 (64)	6 (35)	
Less urgent	34 (52)	15 (44)	19 (59)		19 (49)	8 (36)	11 (65)	
Employment status, n (%)				0.98				0.74
Yes	4 (6)	2 (6)	2 (6)		3 (8)	2 (9)	1 (6)	
No	65 (94)	33 (94)	32 (94)		37 (93)	21 (91)	16 (94)	
Education, n (%)				0.80				0.89
High school or less	56 (81)	28 (80)	28 (82)		31 (78)	18 (78)	13 (76)	
Some college or more	13 (19)	7 (20)	6 (18)		9 (22)	5 (22)	4 (24)	
Percent seeing provider within 30 days, n (%)	--	--	--	--	28 (70)	17 (74)	11 (65)	0.53
Percent seeing provider within 2 weeks, n (%)	--	--	--	--				0.73
Yes					13 (39)	7 (37)	6 (43)	
No					20 (61)	12 (63)	8 (57)	
Mean number of providers seen within 30 days ± SD	--	--	--	--	1.7 ± 1.6	1.7 ± 1.6	1.6 ± 1.7	0.82

* The following 11 chronic conditions were measured in this count: heart attack, cancer, angina, diabetes, congestive heart failure, arthritis, stroke, depression, high blood pressure, atrial fibrillation, chronic obstructive pulmonary disease.

** Categorized as high acuity (ESI=1, 2) or less urgent (ESI= 3, 4, 5).

**Table 2:** Unadjusted average patient activation measure (PAM) scores for the intervention and usual care groups during the baseline and follow-up periods, intention to treat (n=40).

	Usual Care (n=17)			Intervention (n=23)				
				
	Baseline	Follow-Up	Difference	Baseline	Follow-Up	Difference	β	p-value
PAM Score	59.976	55.335	−4.64	68.013	65.243	−2.77	1.87	0.043
