# Supplementary material for: Feasibility of an ED-to-Home Intervention to Engage Patients: A Mixed-Methods Investigation
Source: West J Emerg Med. 2017 Apr 19;18(4):743–51. doi: 10.5811/westjem.2017.2.32570 (PMC5468082; doi:10.5811/westjem.2017.2.32570)
Supplement: Supplementary file 1 [file wjem-18-743-s001.docx]

**Supplementary Appendix.** Sample In-depth Interview Questions.

1. Tell me a little about your recent experience in the emergency room?
2. What did you do before going to the emergency room? Did you try to see your regular doctor?
3. How did you decide to go to the emergency room? What kinds of symptoms were you having?
4. What kind of follow-up care did you receive from your regular doctor?
5. What kinds of things do you think we can do to help people better manage their medical conditions so they don’t have to come to the emergency room?

**Additional Sample Questions for Intervention Group**

1. Tell me about [coach’s name], the coach you have been working with for the past month.
2. What kinds of things did [coach’s name] discuss with you?
3. What was most helpful about the things [coach’s name] talked with you about?
